# Deep Learning and Vision Transformer for Medical Image Analysis

**DOI:** 10.3390/jimaging9070147

**Published:** 2023-07-21

**Authors:** Yudong Zhang, Jiaji Wang, Juan Manuel Gorriz, Shuihua Wang

**Affiliations:** 1 School of Computing and Mathematical Sciences, University of Leicester, Leicester LE1 7RH, UK; jw933@le.ac.uk (J.W.); shuihuawang@ieee.org (S.W.); 2 Department of Signal Theory, Networking, and Communications, University of Granada, 52005 Granada, Spain; gorriz@ugr.es

Artificial intelligence (AI) refers to the field of computer science theory and technology [1] that is focused on creating intelligent machines capable of simulating human intelligence [2]. AI systems [3] are designed to perform tasks that typically require human intelligence [4], such as perception, learning, reasoning [5], problem-solving [6], decision-making [7], etc.

Machine learning (ML) [8] is a subfield of AI that encompasses algorithms and statistical models, enabling computer systems to automatically learn from data, identify patterns, and make predictions or decisions without being explicitly programmed [9]. It involves the development of mathematical models and algorithms [10] that allow machines to iteratively process and analyze large datasets, learn from examples or experiences, and improve their performance over time. By leveraging ML theories and techniques [11], computers can discover complex patterns, extract meaningful insights, and generate reliable predictions, making ML a powerful tool for various applications in fields such as finance, smart healthcare [12], the Internet of Things [13], natural language processing (NLP) [14], recommendation systems, etc.

Deep learning (DL) is a specialized branch of ML that focuses on the development and training of artificial neural networks with multiple layers of interconnected nodes [15], which are known as deep neural networks. It enables computers to automatically learn hierarchical representations of data, allowing for the extraction of intricate patterns and features from complex datasets [16]. DL leverages the power of large-scale computing and vast amounts of data [17] to enable neural networks to perform sophisticated tasks, such as image and speech recognition, NLP, and even autonomous decision-making. By emulating the structure and functionality of the human brain, DL has revolutionized AI by significantly enhancing the accuracy and performance of various applications [18] including medical image analysis (MIA) [19], while also demanding substantial computational resources.

Transformers are a revolutionary DL method that have greatly impacted the field of NLP. They are an example of a neural network model designed to process sequential data, such as sentences or paragraphs, by leveraging attention mechanisms. Unlike traditional recurrent neural networks (RNNs) [20] that process input sequentially, transformers [21] employ a parallelized approach, allowing for more efficient and scalable computation. By focusing on the relationships and dependencies between different words or tokens within a sequence, the transformer model excels at tasks like machine translation, text generation, sentiment analysis, and language understanding [22]. Transformers’ self-attention mechanisms enable them to capture contextual information effectively, resulting in state-of-the-art performance on a wide range of NLP benchmarks and applications. Transformers have become the foundation for many advanced language models, such as BERT, ChatGPT [23], and T5, and have significantly advanced the capabilities of language understanding and generation systems. Vision transformers (ViTs) [24] are an adaptation of the classical transformer architecture that apply self-attention mechanisms to process image data [25], making them an exemplary powerful model for tasks in computer vision, showcasing the extension of transformers’ effectiveness beyond NLP. Figure 1 shows the relationship between AI, ML, DL, and Transformers.

Medical image analysis (MIA) [26] is an important field of application for AI. MIA involves a series of common procedures [27], starting with image acquisition, wherein medical imaging modalities capture anatomical or functional information. The acquired images then undergo preprocessing techniques [28] to correct artifacts, enhance quality, and standardize the data. Next, segmentation methods [29] are employed to separate and identify specific structures or regions of interest within the images. Registration techniques [30] are applied to align multiple images or different modalities for spatial correspondence.

Feature extraction algorithms [31] extract relevant quantitative or qualitative information from the segmented regions for subsequent analysis. Classification methods [32] are then utilized to classify the extracted features, enabling the identification of diseases or conditions. Visualization techniques [33] help in the interpretation and display of the analysis results for clinicians and researchers. Localization methods [34] precisely determine the spatial location of abnormalities or structures within the images, aiding in diagnosis and treatment planning. These procedures, shown in Figure 2, collectively contribute to the comprehensive analysis and interpretation of medical images, ultimately facilitating improved patient care and medical research [35].

DL for MIA faces several challenges. Acquiring a sufficient quantity of high-quality annotated medical images can be challenging due to privacy concerns, limited availability, and the time-consuming process of manual annotation [36]. DL and ViT models often require a large amount of labeled data to achieve optimal performance, and this data may be limited for rare diseases [37] or specific subpopulations. Further, DL and ViT models typically have a large number of parameters, making them demanding and in need of substantial computational resources [38] for training and inference.

## Figures and Tables

**Figure 1:**
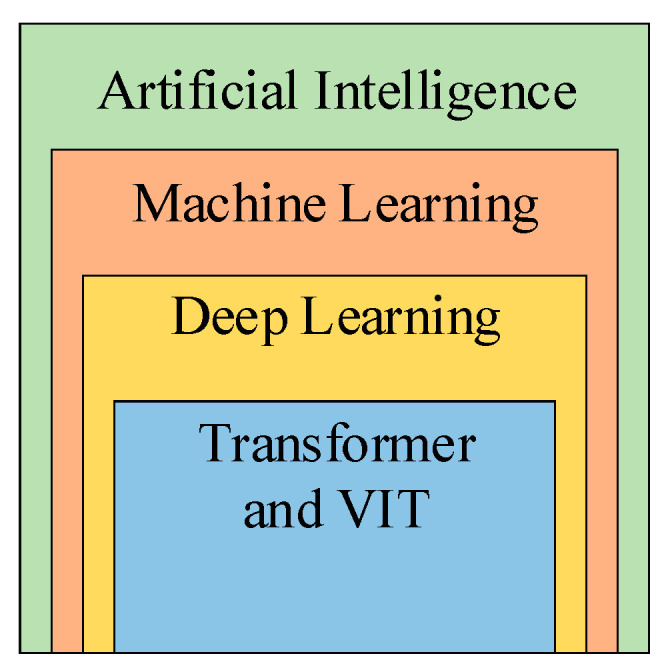
Relationship between AI, ML, DL, and Transformers.

**Figure 2:**
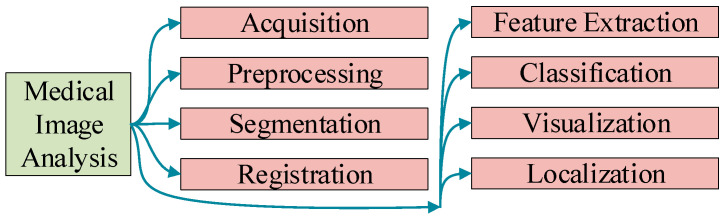
Eight common procedures in medical image analysis.
